# Study on influencing factors and mechanism of removal of Cr(VI) from soil suspended liquid by bentonite-supported nanoscale zero-valent iron

**DOI:** 10.1038/s41598-020-65814-3

**Published:** 2020-06-01

**Authors:** Shichao Liu, Hongjun Gao, Rui Cheng, Yujun Wang, Xiulan Ma, Chang Peng, Zhonglei Xie

**Affiliations:** 1 College of Resources and Environment, Jilin Agricultural University, Changchun, 130118 China; 2 Institute of Agricultural Resources and Environment, Jilin Academy of Agricultural Sciences (Northeast Agricultural Research Center of China), Changchun, 130033 China; 3 Key Laboratory of Aquatic Ecology and Environment, Northeast Institute of Geography and Agroecology, Chinese Academy of Sciences, Changchun, 130102 China; 4 University of Chinese Academy of Sciences, Beijing, 100049 China; 5 College of Plant Science, Jilin University, Changchun, 130062 China; 6 College of Construction Engineering, Changchun Sci-Tech University, Changchun, 130600 China

**Keywords:** Environmental sciences, Environmental chemistry, Pollution remediation

## Abstract

In order to clarify the mechanism and effect of bentonite-supported nanoscale zero-valent iron (nZVI@Bent) on Cr(VI) removal in soil suspended liquid, nZVI@Bent was prepared by liquid-phase reduction method in this research. A number of factors, including the mass ratio of Fe^2+^ to bentonite during preparation of nZVI@Bent, nZVI@Bent dosage, soil suspended liquid pH value and reaction temperature were assessed to determine their impact on the reduction of Cr(VI) in soil suspended liquid. The nZVI@Bent was characterized by scanning electron microscope (SEM), X-ray diffraction (XRD) and X-ray photoelectron spectroscopy (XPS) to analyze the mechanism of removal of Cr(VI) from the soil. The results showed that the temperature of soil suspended liquid had a significant effect on the removal efficiency. Calculated by the Arrhenius formula, nZVI@Bent removes Cr(VI) from the soil suspended liquid as an endothermic reaction with a reaction activation energy of 47.02 kJ/mol, showed that the reaction occurred easily. The removal of mechanism Cr(VI) from the soil by nZVI@Bent included adsorption and reduction, moreover, the reduction process can be divided into direct reduction and indirect reduction. According to XPS spectrogram analysis, the content of Cr(III) in the reaction product was 2.1 times of Cr(VI), indicated that the reduction effect was greater than the adsorption effect in the process of Cr(VI) removal. The experiment proved that nZVI@Bent can effectively remove Cr(VI) from soil suspension, and can provide technical support for repairing Cr(VI)-polluted paddy fields.

## Introduction

Chromium (Cr) is a heavy metal element that exists on the earth, mostly in the two valence states of Cr(III) and Cr(VI). Under certain conditions, Cr(VI) will be reduced to Cr(III) by reducing substances in the soil. Different environmental pollution effects of chromium in different valence states. Cr(VI) can be inhaled with dust to cause respiratory diseases. Oral administration can also cause digestive tract corrosion. After long-term exposure to the high concentration of Cr(VI), gene expression may be changed by destroying DNA, protein and lipid, which can cause cancer in severe cases^1,2^. However, the toxicity of Cr(III) is much lower than that of Cr(VI), moreover, Cr(III) was an essential trace element in biological activities, and low concentration of Cr(III) was beneficial to organisms^3–6^. Compared with Cr(VI), Cr(III) is more stable in the environment and difficult to migrate. Many industries containing chromium pollution such as metallurgy, tannery and electroplating and so on had sprung up in recent years. The discharge of wastewater in these industries lead to increasing the concentration of chromium in the cultivated soil environment, meanwhile, the land near factories was also polluted by a large accumulation of chromium slag and chromium dust^7–9^. It is almost impossible to remove Cr from the soil, so it is the focus of this study to transform the highly toxic and easily migrated Cr(VI) into low toxic and stable Cr(III) can be an effective way to reduce the toxicity of Cr in the soil^10^. According to previous literature, the commonly used existing remediation methods for heavy metal pollution in soil include physical remediation, phytoremediation, and microbial remediation^11,12^. However, some disadvantages that occurred in the above remediation process were high cost, long cycle and possible damage to the soil ecosystem. Among these approaches, solidification/stabilization technology is a mature technology to become an alternative technology due to its lower-cost and less secondary pollution and eco-friendly. Based on these characteristics, the solidification/stabilization technology is widely used in Cr(VI) pollution treatment.

Nanoscale zero-valent iron (nZVI) has been considered to be a good repair agent for solidification/stabilization technology in recent years due to its large reaction area and strong reactivity^13–15^. Take advantage of these characteristics of nZVI, the Cr(VI) in soil can be reduced to Cr(III). The research illustrated that when the dosage of nZVI was 5 g/L, the removal rate of Cr(VI) could reach 99% after 50 days^16^, although the removal rate was high but the reaction period was too long. Previous researches proved that nZVI had a much higher ability to remove Cr(VI) from the soil than ferrous sulfate^17^. However, the common nZVI has too high activity and there is a large electrostatic force on the surface, which leads to the agglomeration of nZVI^18,19^, greatly reducing its reaction area and affecting its reactivity.

In order to avoid nZVI agglomeration, much porous materials support was used to improve nZVI’s dispersion, increase reactive sites, remove pollutants synergistically, and improve the mobility of nZVI^20–23^. Porous materials made up for the shortcomings of nZVI and greatly improved the ability of nZVI to remove pollutants. Carboxymethyl cellulose (CMC), layered dihydroxides (LDH), graphene were currently studied as supporting materials^24–26^, however, the wide application of these supporting materials was limited because of secondary pollution, rare materials and high cost. The main component of bentonite is montmorillonite, a kind of silicate with a flaky structure. Bentonite has many excellent characteristics such as non-toxicity, low cost, abundant reserves and eco-friendly. As supporting materials, bentonite has good adsorbability and hence can absorb a number of nZVI to load on its own surface. In addition, bentonite also has suspension and dispersion^27^, which can boost nZVI particles dispersion and reduce agglomeration, these properties simultaneously make the newly prepared composite nZVI have good adsorption properties. According to these characteristics, bentonite can be used as a supporting material to load nZVI to improve the ability of nZVI to remove pollutants. Although previous articles had reported the removal of Cr(VI) from water by bentonite loaded with nanoscale -valent iron, few articles had been published on the removal of Cr(VI) from soil suspended liquid.

The aim of this study was to explore the removal ability of nZVI@Bent to Cr(VI) in soil suspended liquid including two aspects: (1) to explore the effect of different influencing factors on the removal of Cr(VI) from soil suspended liquid by nZVI@Bent; (2) to explore the reaction mechanism of nZVI@Bent removing Cr(VI) from soil extract. This study can provide a theoretical basis and technical support for the treatment of Cr(VI)-polluted paddy fields.

## Results and discussion

### Effect of Fe^2+^ to Bentonite Mass Ratio on Reduction Efficiency of Cr(VI)

**Figure 1:**
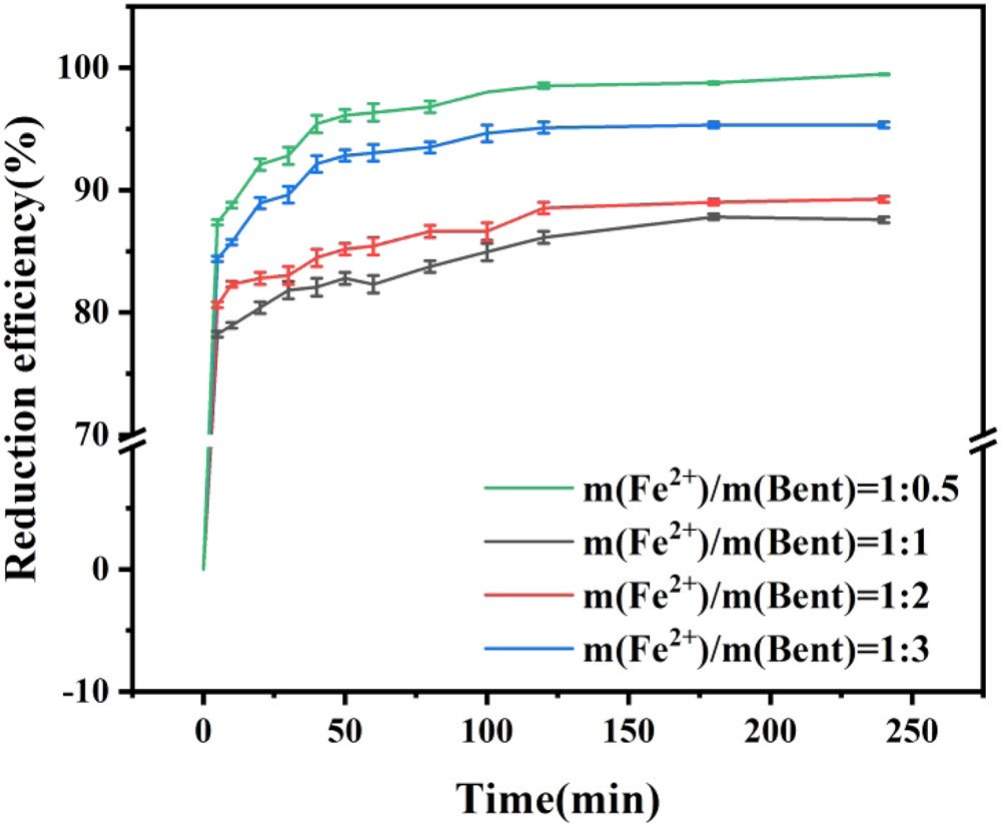
Effect of mass ratio of Fe^2+^ to bentonite on removal efficiency.

As shown in Fig. 1, nZVI@Bent particles had great effects on Cr(VI) removal in contaminated soil suspended liquid. When the reaction time was 240 minutes, the removal efficiency of Cr(VI) from soil suspended liquid by nZVI@Bent particles with the mass ratio of Fe^2+^ to bentonite of 1:3 and 1:2 was only 87.58% and 89.26%, respectively, which efficiency was not satisfactory. However, when the mass ratio of Fe^2+^ to bentonite increased to 1:1 and 1:0.5, the removal efficiency of Cr(VI) from soil suspended liquid could reach 95.33% and 99.46% respectively. The results showed that the efficiency of nZVI@Bent particles remove Cr(VI) from soil suspended liquid increased with the increase of the mass ratio of Fe^2+^ to bentonite. The experimental result of this paper was similar to the finding of a previous study^26^, in which the removal efficiency of phosphorus in the water rose with the increase of the nZVI in graphene supported nanoscale zero-valent iron (G-nZVI) materials. This may be because that when the quality of Fe^2+^ was lower in the preparation process, nZVI particles in nZVI@Bent were less and too dispersed on bentonite, even some of nZVI had been coated by bentonite. These reasons lead to the reduction of the number of molecules with reactive activity in nZVI@Bent. Cr(VI) cannot combine with reactive molecules, resulting in a decrease in the ability of nZVI@Bent to remove Cr(VI) from soil suspended liquid.

The reaction rate is another key indicator in evaluating the ability of nZVI@Bent remove Cr(VI) from soil suspended liquid. Apparent rate constant k_obs_ indicate the reaction rate of nZVI@Bent to remove Cr(VI) from soil suspended liquid. The larger the apparent rate constant, the faster the reaction rate is^28^. Figure 1 showed that the removal rate of Cr(VI) changed slightly after 60 min, so the data of the first 60 min in this experiment was selected to use ln(c_t_/c_0_) for linear fitting of t to obtain the apparent rate constant k_obs_ of the reaction. The fitting results were shown in Table 1, when the mass ratio of Fe^2+^ to bentonite was 4 different treatments, ln(c_t_/c_0_) had a very significant linear relationship with t. The apparent rate constant k_obs_ increased with the increasing proportion of Fe^2+^ in the preparation process. This illustrated that the removal rate of Cr(VI) from the soil suspended liquid by nZVI@Bent was promoted with the increase of the proportion of Fe^2+^ mass in the preparation process. This was probably attributed to the increase of nZVI content in nZVI@Bent caused a large amount of Cr(VI) to combine with nZVI and react at the initial stage of the reaction, thus greatly increased the reaction rate.

**Table 1:** Apparent rate constant k_obs_ of different Fe^2+^ and bentonite mass ratios.

Fe^2+^ and bentonite mass ratios	k_obs_(min^−1^)	r
1:3	0.0041	0.9298^**^
1:2	0.0049	0.9708^**^
1:1	0.0155	0.9787^**^
1:0.5	0.0240	0.9836^**^

Note: ^**^ indicates an extremely significant correlation (*p* ≤ 0.001).

### Effect of nZVI@Bent dosage on the reduction efficiency of Cr(VI)

**Figure 2:**
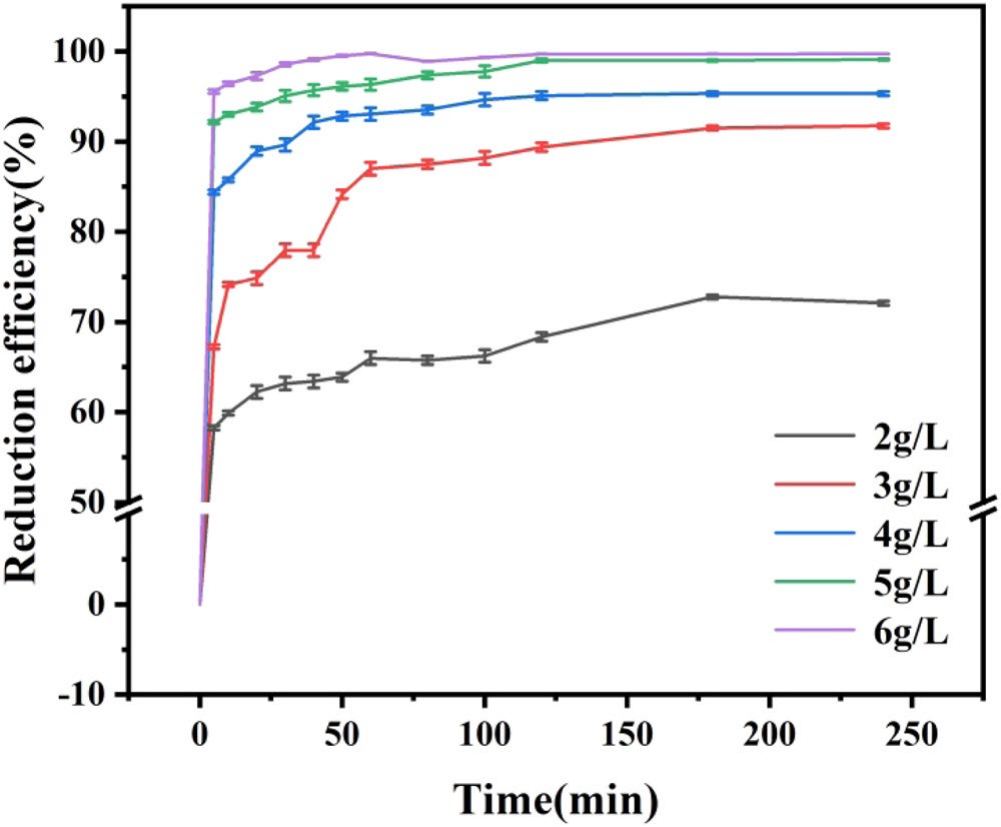
Effect of nZVI@Bent dosage on removal efficiency.

Figure 2 showed the effect of nZVI@Bent dosage on removal efficiency. When the dosage of nZVI@Bent was 2.00, 3.00, 4.00, 5.00 and 6.00 g/L, the removal efficiency of Cr(VI) in soil suspended liquid was 72.08%, 91.74%, 95.33%, 99.10% and 99.75%, respectively. Through comparison, the removal efficiency of Cr(VI) was increased by 27.67% when the dosage increased from 2.00 g/L to 6.00 g /L. The dosage of nZVI@Bent had a significant effect on the removal efficiency of Cr(VI) in soil suspended liquid. The efficiency of nZVI@Bent to remove Cr(VI) in soil suspended liquid increased when the dosage increased. The trend of removal efficiency of Cr(VI) in soil suspended liquid was the same as that of nanoscale valent iron–copper bimetal (nZVI/Cu) with different dosage reported by previous study^28^. This was because the number of nZVI with reactivity and the reaction area will increase as the content of nZVI@Bent in soil suspended liquid increase. More Cr(VI) will be combined with nZVI@Bent and removed by the reaction, and the removal efficiency of Cr(VI) in soil suspended liquid will increase.

The experimental data of the first 60 minutes were selected to make a linear fitting of t with ln(c_t_/c_0_) (Table 2). The standard deviation showed that the ln(c_t_/c_0_) of Cr(VI) reaction in soil suspended liquid with different dosage had an extremely significant linear correlation with t. Moreover, the apparent rate constant of Cr(VI) k_obs_ in soil suspended liquid was increased with the increase of nZVI@Bent. Previous researcher^29^ found that the apparent rate constant k_obs_ of removal of Cr(VI) from wastewater by nZVI increased with the increase of the amount of nZVI added to the wastewater containing Cr(VI), which was a similar result with this study. Similar results further proved that the removal rate of Cr(VI) in soil suspended liquid by nZVI@Bent was promoted with the nZVI@Bent dosage increased. It can be explained that with the increased of nZVI@Bent particles, reactive molecules also increased. At the beginning of the reaction, a large amount of Cr(VI) will be attached to nZVI@Bent particles and removed, resulted in an increase in the reaction rate.

**Table 2:** Apparent rate constants k_obs_ of reactions with different nZVI@Bent dosages.

nZVI@Bent dosages (g/L)	k_obs_ (min^−1^)	R
2.00	0.0032	0.9557^**^
3.00	0.0145	0.9577^**^
4.00	0.0155	0.9787^**^
5.00	0.0167	0.9548^**^
6.00	0.0532	0.9941^**^

Note: ** indicates an extremely significant correlation (p ≤ 0.001).

### Effect of pH of soil suspended liquid on reduction efficiency of Cr(VI)

**Figure 3:**
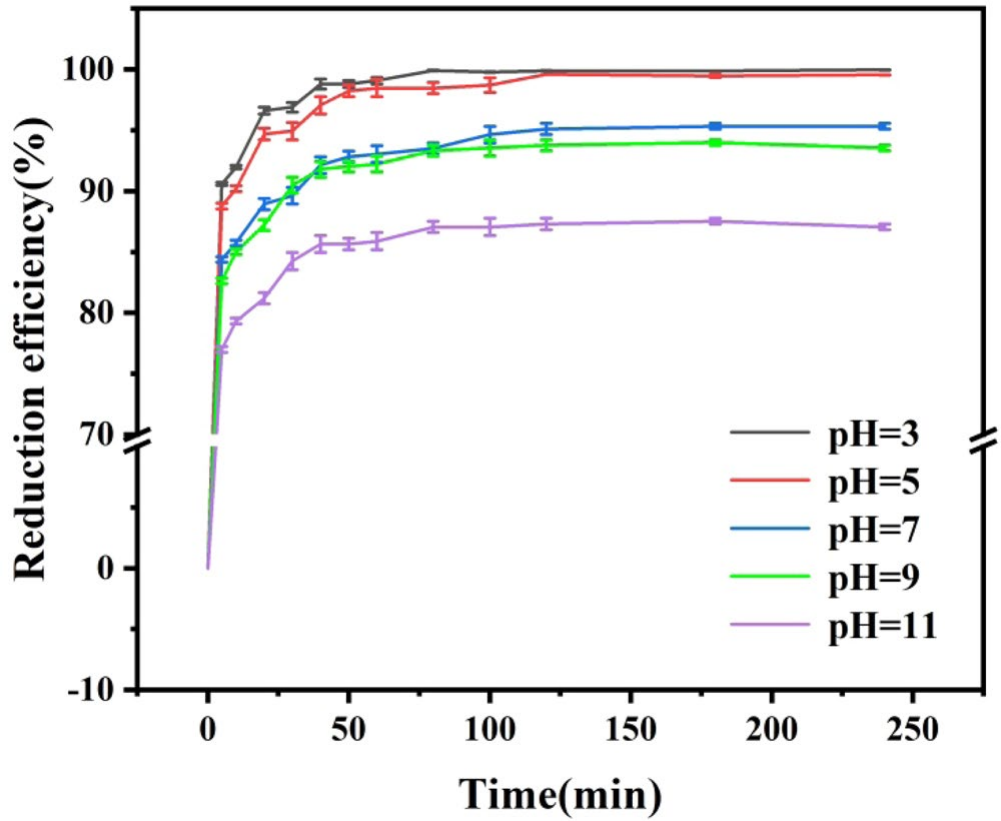
Effect of pH of soil suspended liquid on removal efficiency.

Figure 3 showed that the removal efficiency of Cr(VI) in soil suspended liquid was 99.87%, 99.59%, 95.10%, 93.75% and 87.29%, when the reaction at pH 3, 5, 7, 9 and 11, respectively. With the increase of pH value, the removal efficiency of Cr(VI) in the soil suspended liquid significantly decreased, which indicated that nZVI@Bent was beneficial to the removal of Cr(VI) in the soil suspended liquid under acidic environment. The removal efficiency of nZVI@Bent decreased when the soil suspended liquid changed from acidic to alkaline. Zhu *et al*.^30^ also found that the removal efficiency of Cr(VI) by nanoscale valent iron/Ni bimetal materials (nZVI/Ni) decreased from 99.84% to 52.63% when the pH of soil suspended liquid increased from 5 to 7 and other conditions remained unchanged. This was because nZVI@Bent consumes H^+^ in the process of removing Cr(VI), while in an acidic environment, there was a large amount of free H^+^ in the soil suspended liquid. A large amount of H^+^ can promote the reaction direction of Cr(VI) removal from nZVI@Bent. Moreover, under the acidic environment, the Fe_2_O_3_ and FeOOH oxide layer attached to the surface of nZVI@Bent will be corroded to expose more reaction sites. More Cr(VI) contacted reaction sites and then were removed, so as to improve the efficiency of removing Cr(VI) from the soil. However, when the soil suspended liquid was alkaline, Fe^2+^ and Fe^3+^ will form a large amount of Fe(OH)_X_(X = 2,3) accumulated with excessive OH^−^ in the suspended liquid. The Fe(OH)_X_(X = 2,3) formed a layer of iron hydroxide film covered the surface of nZVI@Bent prevented the contact of nZVI@Bent with Cr(VI), resulted in a decline of the reduction rate.

The nZVI@Bent particles were separated from the soil suspended liquid and scanned by SEM after reaction under different pH conditions. It was found that as the pH dropped from 11 to 3, more flaky structures appeared on the surface of the nZVI@Bent particles, and the particle size of the material became larger. A small amount of nZVI less than 100 nm will be found on the surface of nZVI@Bent particles under alkaline conditions. Most nZVI particles showed a flake structure under acidic conditions. Under alkaline conditions, nZVI was corroded by OH^−^ in the environment to generate Fe(OH)_3_ or FeOOH. The iron hydroxide film made nZVI difficult to react with Cr(VI) in suspended liquid, thus retaining nZVI itself morphology. However, as pH decreased, most nZVI reacted with Cr(VI) in soil suspended liquid, causing nZVI to be oxidized into a flake structure exceeding 100 nm, thus increasing nZVI’s particle size.

When the pH of soil suspended liquid was 3, 5, 7, 9 and 11, the experimental data of the first 60 minutes were selected to fit t linearly with ln(c_t_/c_0_) (Table 3). The linear relationship between ln(c_t_/c_0_) and t was very significant (0.9683^**^, 0.9880^**^, 0.9787^**^, 0.9553^**^, and 0.9453^**^). As the pH of the soil suspended liquid increased, the apparent rate constant k_obs_ of nZVI@Bent removing Cr(VI) became smaller. This was because there was a layer of hydroxide of Fe on the surface of nZVI@Bent particle under alkaline conditions, which hindered Cr(VI) from reacting with nZVI, thus reduced the reaction rate.

**Table 3:** Apparent rate constants k_obs_ of pH of different soil suspended liquid.

pH of soil suspended liquid	k_obs_ (min^−1^)	r
3	0.0442	0.9683^**^
5	0.0373	0.9880^**^
7	0.0155	0.9787^**^
9	0.0154	0.9553^**^
11	0.0092	0.9453^**^

Note: ** indicates an extremely significant correlation (p ≤ 0.001).

### Effect of reaction temperature on reduction efficiency of Cr(VI)

**Figure 4:**
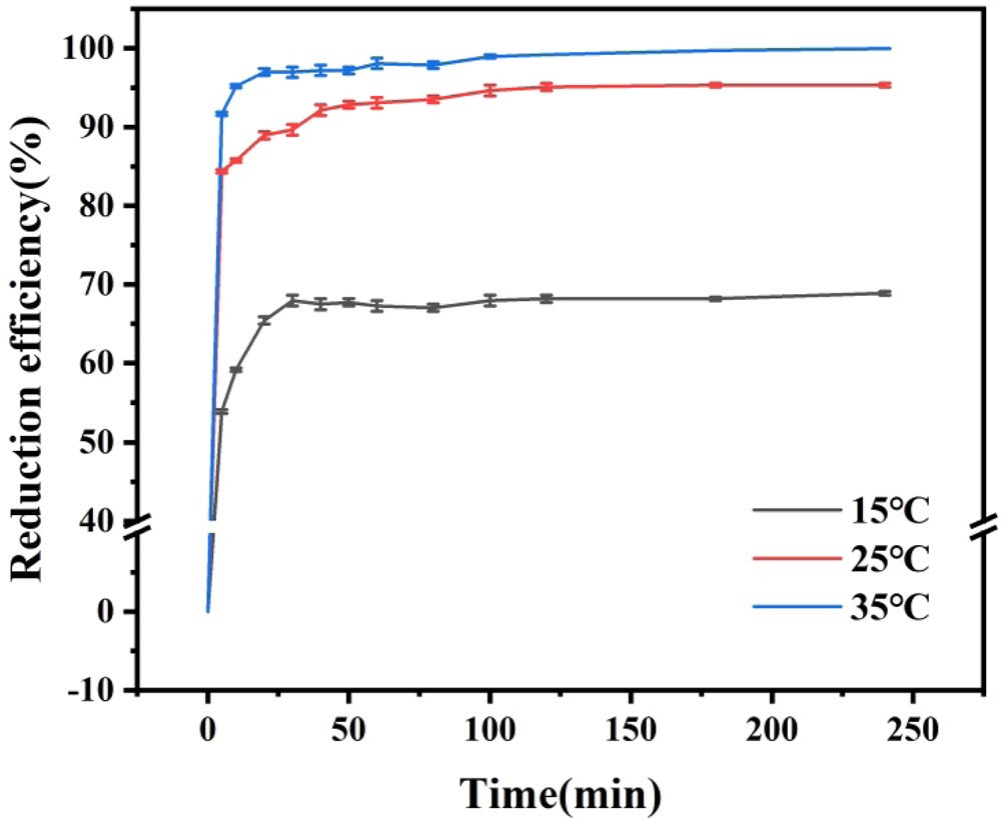
Effect of reaction ambient temperature on removal efficiency.

As shown in Fig. 4, at 15, 25 and 35 °C, Cr(VI) removal efficiency in the soil suspended liquid after reaction equilibrium were 68.88%, 95.33% and 99.95%, respectively. The removal efficiency of Cr(VI) from soil suspended liquid increased with the increase of temperature.

The nZVI@Bent particles reacted at different temperatures were separated from the suspension for SEM scanning. With the increase of temperature, the surface of nZVI@Bent particles had more flaky structures, and the material size became larger. Under the condition of low temperature, there were fewer active particles on the nZVI@Bent surface. nZVI@Bent cannot react with Cr(VI) in soil suspended liquid, thus retaining its own structure. However, with the increase of temperature, more particles gain energy and become active particles to react with Cr(VI) in suspended liquid, and more nZVI particles react to form large flake structures, thus increasing their particle sizes.

Table 4 showed the fitting of the reaction of nZVI@Bent for removing Cr(VI) from soil suspended liquid at different temperatures with a pseudo-first-order model reaction. The results showed that the reaction conformed to the pseudo-first-order kinetics model when the reaction ambient temperature was 15, 25 and 35 °C respectively. The apparent rate constant k_obs_ of nZVI@Bent for removing Cr(VI) from soil suspended liquid increased with the increase of reaction temperature. Due to the reason that the energy in reaction system increased with the temperature rose, there were more reactant molecules and higher reaction rates; the rate of remove Cr(VI) from soil solution by nZVI@Bent was boosted by the rising of reaction environment temperature.

**Table 4:** Apparent rate constant k_obs_ at different reaction temperatures.

Different reaction temperatures (°C)	k_obs_ (min^−1^)	r
15	0.0057	0.8132^**^
25	0.0155	0.9787^**^
35	0.0202	0.9707^**^

Note: ** indicates an extremely significant correlation (p ≤ 0.001).

According to the Arrhenius formula, the equation of *ln k*_*obs*_ = −5655.3 $\frac{1}{T}$ + 14.569 was obtained by drawing curves of apparent rate constants with temperature. The apparent activation energy of removing Cr(VI) from soil suspended liquid by nZVI@Bent is 47.02 kJ/mol, and the preexponential factor k_0_ was 2.124 × 10^6^ min^−1^. It indicated that the Cr(VI) reaction in soil suspended liquid removed by nZVI@Bent was an endothermic reaction. When the temperature of the reaction system increased, the reaction would be moved forward, resulted in more Cr(VI) reacted with nZVI@Bent and be removed.

### Optimal conditions for removal of Cr(VI) by nZVI@Bent

At the ratio 1:0.5 of Fe to bentonite in nZVI@Bent particles, nZVI@Bent had the highest removal rate of Cr(VI) in soil suspended liquid. At the dosage 5 g/L and 6 g/L of nZVI@Bent, the removal rate of Cr(VI) reached more than 99%. Considering the economic factor of practical application, the better dosage of nZVI@Bent was 5 g/L. Under the pH 3 and 5 of the reaction environment, the removal rate of Cr(VI) reached more than 99%. Considering the higher acidity in the soil will limit the growth of crops, the pH 5 of the reaction environment was suitable condition. At the temperature 35 °C, the removal rate of Cr(VI) was the highest. Consequently, the optimum condition for nZVI@Bent to remove Cr(VI) from soil suspended liquid was the ratio 1:0.5 of Fe to bentonite in nZVI@Bent particles, the dosage 5 g/L, the reaction environment pH 5 and the reaction environment of 35 °C.

Shi^31^, Wang^32^ conducted a study of the removal effect of nZVI@Bent on Cr(VI) in water, which is relatively easy compared with the removal of paddy Cr(VI) in this paper. However, the optimal conditions for the removal of Cr(VI) by nZVI@Bent prepared by different scholars can be compared. Table 5 showed that different scholars used the same supported material, the optimum removal conditions of different removal materials were different because of the different Fe to bentonite mass ratio controlled by each scholar during the preparation process. Although the nZVI@Bent material prepared by Shi *et al*.^31^ had the advantages of low dosage and low iron content, it required low pH in the environment and high reaction temperature, which brought similar problems of high cost in practical application. Although the nZVI@Bent material prepared by Wang *et al*.^32^ had the advantage of low dosage, the high iron content of the material will make the nZVI@Bent had a large amount of iron left in the water after removing pollutants, resulting in secondary pollution. Moreover, nZVI@Bent prepared by Wang *et al*.^32^ required a high acidity environment (pH 2), which has strict requirements on the pH of the wastewater. The nZVI@Bent material prepared in this study had the disadvantage of large dosage, but it had the advantages of appropriate iron content, close to neutral reaction environment and relatively low reaction environment temperature. The advantages of nZVI@Bent in this study will avoid secondary iron pollution after the reaction, acidification of paddy fields and high removal cost. So this research is meaningful and can bring technical support for actual production and life.

**Table 5:** Optimum reaction conditions for the removal of Cr(VI) from Bentonite-supported nanoscale Zero-Valentiron prepared by different scholars.

Author	Fe to Bentonite Mass Ratio	Dosage (g/L)	pH	Temperature (°C)
Shi^31^	1:1	4	2	40
Wang^32^	1:0.04	3	2	—
This article	1:0.5	5	5	35

Previous studies had also used different supported materials to prepare composite nZVI, but they had some drawbacks in their application to the removal of Cr(VI). For example, MCM-41-supported nanoscale zero-valent iron prepared by Petala *et al*.^33^ needed to be performed in a low pH environment when removing Cr(VI), which will cause the harm of water acidification. The attapulgite-supported nanoscale zero-valent iron prepared by Zhang *et al*.^34^ required a long contact time to remove Cr(VI), which greatly affected the removal efficiency of Cr(VI)-containing wastewater. Therefore, nZVI@Bent has certain advantages in removing Cr(VI).

### Removal of Cr(III) by nZVI@Bent

As can be seen from the above, the optimum condition for nZVI@Bent to remove Cr(VI) from soil suspended liquid was the ratio 1:0.5 of Fe to bentonite in nZVI@Bent particles, the dosage 5 g/L, the reaction environment pH 5 and the reaction environment of 35 °C. Under these conditions, the removal rate of Cr(III) by nZVI@Bent was 92.36%. Most of Cr(III) was removed and further immobilized free Cr(III), which made Cr more stable and reduced the harm brought by Cr.

### SEM characterization

**Figure 5:**
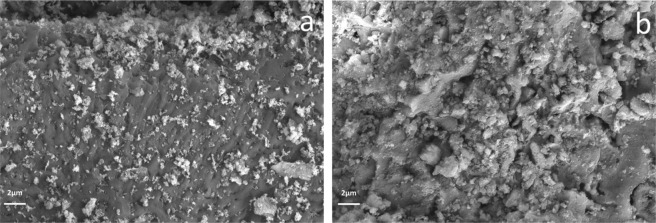
SEM images of nZVI@Bent: (**a**) nZVI@Bent before reaction; (**b**) nZVI@Bent after reaction.

Figure 5(a) showed the structure and morphology of nZVI@Bent’s. The size of nanoscale zero-valent iron particles was 40–80 nm and had been loaded on bentonite and only a small amount of agglomeration occurred. The nanoscale zero-valent iron particles can be dispersed more on bentonite and effectively prevent agglomeration of zero-valent iron nanoparticles. Figure 5(b) was the scanning electron microscope image of nZVI@Bent reacted with Cr(VI) in soil suspended liquid. There were a large number of flake structures on the surface of nZVI@Bent reacted with Cr(VI) in soil suspended liquid. The spherical nZVI of nZVI@Bent was completely oxidized and formed a large number of flake structures after reacted with Cr(VI)-contaminated soil suspended liquid. This was due to the formation of Fe(III)-Cr(III) complexes attached to the nZVI@Bent surface which reacted with Cr(VI) in the soil suspended liquid, and then a large number of Fig. 5(b) flake structures appeared.

### XRD characterization

**Figure 6:**
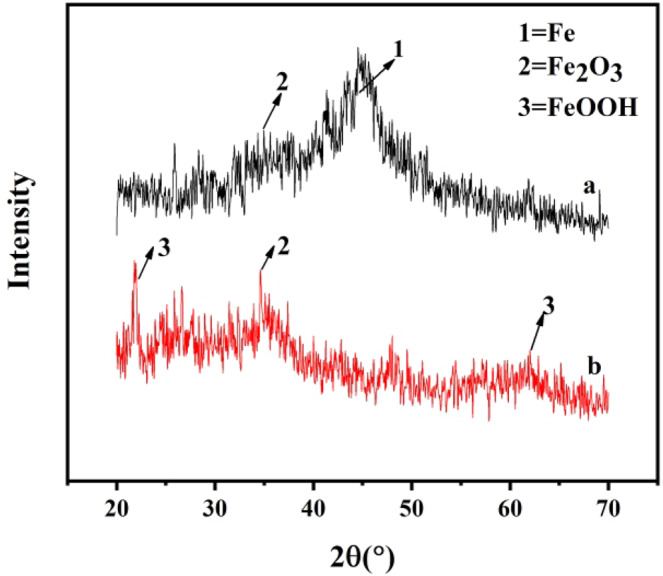
XRD pattern of nZVI@Bent: (**a**) nZVI@Bent before reaction; (**b**) nZVI@Bent after reaction.

X-ray diffraction was used to explore the nZVI@Bent particle characterizations conducted before and after the reaction with Cr(VI). Figure 6(a) showed that there were obvious Fe^0^ peaks and weak Fe_2_O_3_ peaks in freshly prepared nZVI@Bent particles. The obvious Fe^0^ peaks indicated that nZVI had been loaded on bentonite surface. The weak Fe_2_O_3_ peaks indicated that a small amount of Fe_2_O_3_ was attached to the surface of nZVI@Bent. The XRD pattern of biochar supported nanoscale zero-valve iron prepared by Li *et al*.^35^ showed obvious peaks of Fe_2_O_3_ and FeOOH, nZVI supported with biochar will not effectively reduce the oxidation. However, there were no obvious peaks of Fe_2_O_3_ and FeOOH in Fig. 6(a), which indicated that nZVI supported with bentonite can effectively prevent the oxidation of nZVI. Figure 6(b), showed that nZVI@Bent particles had no obvious Fe^0^ peak after reacting with soil suspended liquid containing Cr(VI) pollution, but had obvious Fe_2_O_3_ and FeOOH peaks, it suggested that nZVI had been nearly completely reacted with soil suspended liquid containing Cr(VI) pollution, and most of them had been oxidized to form Fe_2_O_3_ and FeOOH. The reaction equation is as follows:

a $${{\rm{Fe}}}^{0}+2{{\rm{H}}}_{2}{\rm{O}}\to {{\rm{Fe}}}^{2+}+{{\rm{H}}}_{2}+2{{\rm{OH}}}^{-}$$

b $$4{{\rm{Fe}}}^{2+}+{{\rm{O}}}_{2}+4{{\rm{H}}}_{2}{\rm{O}}\to 2{{\rm{Fe}}}_{2}{{\rm{O}}}_{3}+8{{\rm{H}}}^{+}$$

c $$4{{\rm{Fe}}}^{2+}+4{{\rm{H}}}^{+}+{{\rm{O}}}_{2}\to 4{{\rm{Fe}}}^{3+}+2{{\rm{H}}}_{2}{\rm{O}}$$

d $${{\rm{Fe}}}^{3+}+3{{\rm{OH}}}^{-}\to {\rm{Fe}}{({\rm{OH}})}_{3}$$

e $${\rm{Fe}}{({\rm{OH}})}_{3}+3{{\rm{H}}}^{+}\to {\rm{FeOOH}}+{{\rm{H}}}_{2}{\rm{O}}$$

### Mechanism of removing Cr from soil suspended liquid by nZVI@Bent

**Figure 7:**
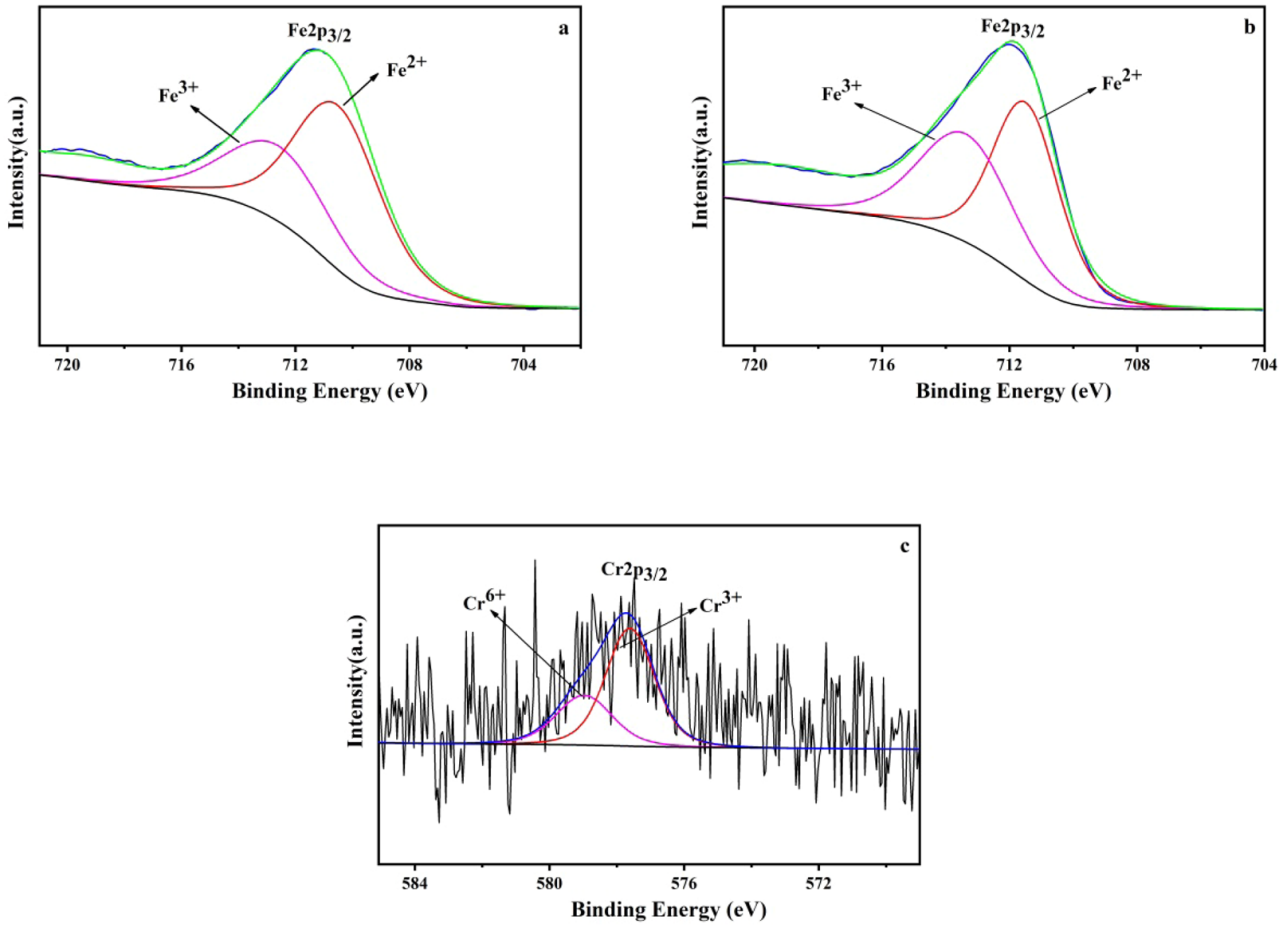
XPS spectra of nZVI@Bent: (**a**) nZVI@Bent-Fe before reaction; (**b**) nZVI@Bent-Fe after reaction; (**c**) nZVI@Bent-Cr after reaction.

Removal of Cr(VI) from soil suspended liquid by nZVI@Bent can be divided into two processes: reduction and adsorption^36^. Cr(VI) in nZVI@Bent reduced soil suspended liquid can be roughly divided into two processes, direct reduction and indirect reduction^10,37,38^. In the direct reduction process, Cr(VI) will be adsorbed on the surface of nZVI@Bent to directly react with the surface containing nZVI to form Cr(III)(Eqs. f,g). nZVI will be oxidized to Fe^2+^ for indirect reduction with Cr(VI): Fe^2+^ reduced Cr(VI) in soil suspended liquid to Cr(III) (Eq. h), and Cr(III) can combine with OH^−^ in soil suspended liquid to form Cr(OH)_3_ (Eq. i), Fe^2+^ was oxidized to Fe^3+^, and Fe^3+^ of the reaction system combines with Cr(III) and free OH^−^ in the soil suspended liquid to form Fe(III)-Cr(III) complex (Eqs. j,k)^39,40^. The peak area of Fe^3+^ increases obviously (Fig. 7(b)), and conversely, the peak area of Fe^2+^ decreased (Fig. 7(a)). The proportion of Fe^2+^ in nZVI@Bent particles decreased from 64.50% before the reaction to 45.57% after the reaction. The proportion of Fe^3+^ increased from 35.50% before reaction to 54.43% after reaction. The change of the peak area of Fe^2+^ and Fe^3+^ further confirmed the removal process of Fe^2+^ and Cr(VI) reaction being oxidized to Fe^3+^ and then forming Fe(III)-Cr(III) complex. With the formation of these complexes and their attachment to the surface of nZVI@Bent, the contact of Cr(VI) with nZVI@Bent will be hindered, resulted in the reduction ability of nZVI@Bent. This hypothesis was also consistent with the fact that the nZVI@Bent reaction rate in batch experiments was very high at the initial stage of the reaction and became slower with the reaction proceeds.

XPS analysis confirmed that there was no material containing Cr in the freshly prepared nZVI@Bent particle. After the reaction with soil suspended liquid containing Cr(VI), the materials containing Cr appeared on the surface of the nZVI@Bent particle (Fig. 7(c)). There was a larger peak area for Cr(III) than that for Cr(VI), and the ratios of Cr(III) and Cr(VI) on the surface of nZVI@Bent particles after the reaction were 67.35% and 32.65%, respectively. It indicated that the reduction of Cr(VI) in the system of nZVI@Bent particles and the soil suspended liquid containing Cr(VI) was the main process compared with the adsorption. The reduction reaction was also more intense and most of the Cr(VI) was removed by the reduction reaction, and the amount of Cr(VI) left in the soil greatly decreased.

The main principle of adsorption concerned with both the large specific surface area of nZVI@Bent, and was more adsorption sites on its surface. Not only Cr(VI) was directly adsorbed by nZVI@Bent in the soil suspended liquid, but also Cr(OH)_3_ and Fe(III)-Cr(III) complexes produced during the reduction process were adsorbed by nZVI@Bent, further reduced soil Cr pollution.

**Figure 8:**
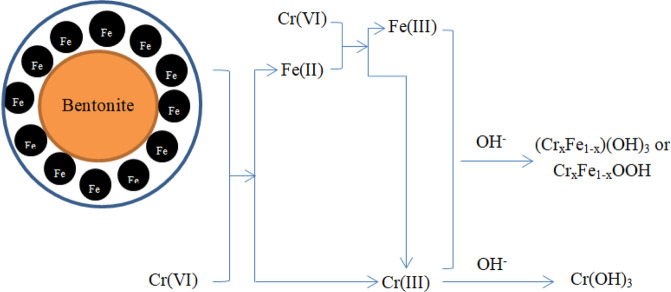
Reduction flow chart of Cr(VI) removal from soil suspended liquid by nZVI@Bent.

Based on the above removal reaction process, the reduction process (Fig. 8) and the reduction process equation of Cr(VI) can be roughly inferred as follows^31,41–43^:

Cr(VI) is reduced to Cr(III) equation:

f $${{\rm{Cr}}}_{2}{{\rm{O}}}_{7}^{2-}+3{{\rm{Fe}}}^{0}+14{{\rm{H}}}^{+}\to 3{{\rm{Fe}}}^{2+}+2{{\rm{Cr}}}^{3+}+7{{\rm{H}}}_{2}{\rm{O}}$$

g $${{\rm{Cr}}}_{2}{{\rm{O}}}_{7}^{2-}+3{{\rm{Fe}}}^{0}+7{{\rm{H}}}_{2}{\rm{O}}\to 3{{\rm{Fe}}}^{2+}+2{{\rm{Cr}}}^{3+}+14{{\rm{OH}}}^{-}$$

h $${{\rm{Cr}}}_{2}{{\rm{O}}}_{7}^{2-}+6{{\rm{Fe}}}^{2+}+14{{\rm{H}}}^{+}\to 6{{\rm{Fe}}}^{3+}+2{{\rm{Cr}}}^{3+}+7{{\rm{H}}}_{2}{\rm{O}}$$

i $${{\rm{Cr}}}^{3+}+3{{\rm{OH}}}^{-}\to {\rm{Cr}}{({\rm{OH}})}_{3}\downarrow $$

Fe(III)-Cr(III) complex formation process:

j $$(1-{\rm{x}}){{\rm{Fe}}}^{3+}+{{\rm{xCr}}}^{3+}+3{{\rm{H}}}_{2}{\rm{O}}\to ({{\rm{Cr}}}_{{\rm{x}}}{{\rm{Fe}}}_{1-{\rm{x}}}){({\rm{OH}})}_{3}\downarrow +3{{\rm{H}}}^{+}$$

k $$(1-{\rm{x}}){{\rm{Fe}}}^{3+}+{{\rm{xCr}}}^{3+}+2{{\rm{H}}}_{2}{\rm{O}}\to {{\rm{Cr}}}_{{\rm{x}}}{{\rm{Fe}}}_{1-{\rm{x}}}{\rm{OOH}}\downarrow +3{{\rm{H}}}^{+}$$

## Conclusions

1. Nanoscale zero-valent iron was loaded on bentonite, and the agglomeration of nanoscale zero-valent iron after loading significantly reduced.
2. The ability of nZVI@Bent particles to remove Cr(VI) from soil suspended liquid increased with the increase of the mass ratio of Fe^2+^ to bentonite, nZVI@Bent particles dosage and the temperature in the reaction environment. The ability of nZVI@Bent particles to remove Cr(VI) from soil suspended liquid decreased with the increase of pH in the reaction environment. Moreover, the reaction temperature had the greatest influence on the ability of nZVI@Bent particles to remove Cr(VI) in soil extract. When the temperature increased from 15 °C to 35 °C, other conditions remained unchanged, the removal rate of Cr(VI) in soil extract increased from 68.88% to 99.95%, and the removal rate increased by 31.07%.
3. When the ratio of Fe^2+^ to bentonite in nZVI@Bent particles was 1:0.5, the dosage was 5 g/L, the reaction environment pH was 5 and the temperature was 35 °C, the removal rate of Cr(VI) in soil suspension by nZVI@Bent was the optimum reaction conditions for the removal of Cr(VI)
4. Removal of Cr(VI) from soil extract contaminated by Cr(VI) by nZVI@Bent can be divided into two processes: reduction and adsorption, and the reduction effect mainly proceeds. The final product of reduction was the complex of Cr(OH)_3_ and Fe(III)-Cr(III).

## Materials and methods

### Chemicals and materials

Ferrous sulfate (FeSO_4_·7H_2_O), sodium borohydride (NaBH_4_), anhydrous ethyl alcohol, potassium dichromate (K_2_Cr_2_O_7_), concentrated sulfuric acid (H_2_SO_4_), phosphoric acid(H_3_PO_4_) were purchased from Beijing Chemical Works of China. Diphenylcarbazide (C_13_H_14_N_4_O) was purchased from the Tianjin Institute of Fine Chemicals Retrocession of China. Sodium bentonite was purchased from Longxin Co. Ltd., Henan, China.

### Synthesis of bentonite-supported nanoscale zero-valent iron

FeSO_4_•7H_2_O and bentonite with a total solid mass of 6.00 g (mass ratio of Fe^2+^ to bentonite is 1:1) were added into the three-necked flask containing 40.00 ml anhydrous ethyl alcohol and 10.00 ml ultra-pure water mixture of the three-necked flask, and stirred 30 min using electric power stirrer. Under the condition of continuous stirred, 100.00 ml NaBH_4_ solution with a concentration of 2 mol/L was added to the three-necked flask at a speed of 30–40 drops per minute. After adding NaBH_4_ droplets, continue stirred the mixture in the flask for 40 minutes (all these processes had been protected by nitrogen). The above mixture was filtered with a vacuum pump to obtain nZVI@Bent particles, which were quickly rinsed with anhydrous ethyl alcohol for 3 times, then dried in a vacuum drying oven at 75 °C for 12 h, and put into a dryer filled with nitrogen for later use.

### Collection of contaminated soil and preparation of soil suspended liquid

The contaminated soil used in this experiment was collected from the vicinity of a chromium slag storage plant in Jilin province, China. After the soil dried, passed 100 mesh sieve for later use. The properties of the soil were as following: pH 5.25, total organic matter content 42.5 g/kg, alkali-hydrolyzed nitrogen content 153.5 mg/kg, available phosphorus content 63.2 mg/kg, available potassium content 101.5 mg/kg, and clay proportion was 19.6%, respectively.

Weigh 1.00 g of contaminated soil into a 50 ml polyethylene centrifuge tube, adding 25.00 ml distilled water, then put centrifuge tube into a water bath oscillator at the temperature of 25 °C and oscillated for 2 hours, and then, taking out for standby, and the pH of soil suspended liquid was adjusted to be 7, and more mud was prepared.

The supernatant filtered through 0.45μm membrane by centrifugation in centrifugal containing mud tube. The concentration of Cr_total_ in the filtrate was analyzed using a flame atomic absorption spectrophotometer (17.21 mg/L), Cr(VI) in the filtrate was measured using the diphenylcarbazide spectrophotometric method (15.68 mg/L) as the initial concentration of Cr(VI) in soil suspended liquid. The initial concentration of Cr(III) (1.53 mg/L) is equal to the initial concentration of Cr_total_ minus the initial concentration of Cr(VI).

### Batch experiments

The conduct batch experiments were conducted by using soil suspended liquid prepared in the above step. The dilute NaOH and H_2_SO_4_ solutions were used to adjust pH values. The effect of mass ratio of Fe^2+^ to bentonite during nZVI@Bent preparation (1:0.5, 1:1, 1:2 and 1:3), nZVI@Bent dosage (2.00, 3.00, 4.00, 5.00 and 6.00 g/L), pH of soil suspended liquid (3, 5, 7, 9 and 11) and reaction temperature (15, 25 and 35 °C) were studied. The reaction solution was separately centrifuged at a reaction time of 5, 10, 20, 20, 30, 40, 50, 60, 80, 100, 120, 180 and 240 min, respectively, and the supernatant was passed through a 0.45μm filter, and the residual concentration of Cr(VI) in the filtrate was measured using the diphenylcarbazide spectrophotometric method to calculate the removal efficiency of Cr(VI).

### Structural characterization

Scanning electron microscope (SEM, SU8010, Hitachi) was used to morphology the surface characteristics of nZVI@Bent particles before and after the reaction. Before the reaction, the test material was freshly prepared nZVI@Bent particles. After the reaction, the test material was freshly prepared nZVI@Bent particles which reacted with the soil suspended liquid containing Cr(VI) at pH = 7 and the reaction temperature was 25 °C.

X-ray diffraction (XRD, D/MAX 2250 V, Rigaku) (Cu, Ka) was used to measure the X-ray diffraction patterns of nZVI@Bent particles before and after the reaction (the same test material as that of SEM). The X-ray diffraction spectra were acquired from 5 to 80 degrees, and the scanning speed was 2°/min.

X-ray photoelectron spectrometer (XPS, ESCALAB 250Xi, Thermo Fischer) was employed to analyze the nZVI@Bent particle surface elemental valence before and after the reaction (the same test material as that of SEM). The X-ray source was Al-kα ray (hv = 1486.6ev) with a working voltage of 12.5 kV.

### Data processing

Removal efficiency of Cr(VI) and Cr(III) from soil suspended liquid by nZVI@Bent:

1 $$\omega ( \% )=\frac{{c}_{0}-{c}_{t}}{{c}_{0}}\times 100 \% $$

where: *ω*(%) is the removal efficiency of Cr(VI) or Cr(III) in the soil suspended liquid by nZVI@Bent, *c*_0_ is the initial concentration of Cr(VI) or Cr(III) in the soil suspended liquid (mg/L), and *c*_*t*_ is the concentration of Cr(VI) or Cr(III) in soil suspended liquid at time t (mg/L).

### Kinetic model

Determining the reduction kinetics of Cr(VI) by nZVI@Bent with pseudo-first-order model^33,44^:

2 $$v=-\,\frac{d{c}_{t}}{dt}={k}_{SA}{a}_{s}{\rho }_{m}{c}_{0}$$

where: *k*_*SA*_ is the specific surface area reaction rate coefficient of the material [L/(min•m^2^)], *a*_*s*_ is the specific surface area of the material (m^2^/g), *ρ*_*m*_ is the concentration of the material (g/L), *k*_*SA*_, *a*_*s*_, and *ρ*_*m*_ are the constants in one reaction and *k*_*SA*_, *a*_*s*_, and *ρ*_*m*_ can be expressed by a pseudo-first-order model apparent rate constant *k*_*obs*_ (min^−1^), integral formula (2) to obtain:

3 $$\mathrm{ln}({c}_{t}/{c}_{0})=-\,{k}_{obs}t$$

apparent activation energy calculation

The apparent activation energy of nZVI@Bent particles for removing Cr(VI) from soil suspended liquid solution contaminated by Cr(VI) was calculated by Arrhenius formula^45^.

4 $$\mathrm{ln}\,{k}_{obs}\,=\,\mathrm{ln}\,{k}_{0}\,-\frac{{E}_{a}}{RT}$$

where: *R* is the molar gas constant (J/mol•K); *T* is the thermodynamic temperature (K); *E*_*a*_ is the apparent activation energy (kJ/mol); *k*_0_ is the preexponential factor (min^−1^).
